# Inborn and experience-dependent models of categorical brain organization. A position paper

**DOI:** 10.3389/fnhum.2015.00002

**Published:** 2015-01-23

**Authors:** Guido Gainotti

**Affiliations:** ^1^Center for Neuropsychological Research and Department of Neurosciences, Institute of Neurology Policlinico Gemelli, Catholic University of RomeRome, Italy; ^2^Department of Clinical and Behavioral Neurology, IRCCS Fondazione Santa LuciaRome, Italy

**Keywords:** category-specific semantic disorders, sensory-motor model of semantic knowledge, domains of knowledge hypothesis, role of experience in the representation of objects, inborn models of conceptual representations

## Abstract

The present review aims to summarize the debate in contemporary neuroscience between inborn and experience-dependent models of conceptual representations that goes back to the description of category-specific semantic disorders for biological and artifact categories. Experience-dependent models suggest that categorical disorders are the by-product of the differential weighting of different sources of knowledge in the representation of biological and artifact categories. These models maintain that semantic disorders are not really category-specific, because they do not respect the boundaries between different categories. They also argue that the brain structures which are disrupted in a given type of category-specific semantic disorder should correspond to the areas of convergence of the sensory-motor information which play a major role in the construction of that category. Furthermore, they provide a simple interpretation of gender-related categorical effects and are supported by studies assessing the importance of prior experience in the cortical representation of objects On the other hand, inborn models maintain that category-specific semantic disorders reflect the disruption of innate brain networks, which are shaped by natural selection to allow rapid identification of objects that are very relevant for survival. From the empirical point of view, these models are mainly supported by observations of blind subjects, which suggest that visual experience is not necessary for the emergence of category-specificity in the ventral stream of visual processing. The weight of the data supporting experience-dependent and inborn models is thoroughly discussed, stressing the fact observations made in blind subjects are still the subject of intense debate. It is concluded that at the present state of knowledge it is not possible to choose between experience-dependent and inborn models of conceptual representations.

## Introduction

Contemporary debates about the foundations of categories and concepts have very ancient roots. Indeed, discussions about the nature of concepts, which have tried to clarify whether or not they are essentially grounded in our senses and in our actions with objects, go back to the early days of Plato and Aristotle (Kiefer and Pulvermüller, 2012; Markie, 2013). According to Plato and other rationalist philosophers concepts are mental entities and are fundamentally distinct from sensory impressions. By contrast, according to Aristotle and other empiricist philosophers all concepts are derived from sensory experiences. The rationalist position has been endorsed in more recent years by Pylyshyn (1973), Fodor (1975), Caramazza et al. (1990) and Patterson and Hodges (2000). These authors proposed the existence of a unitary, abstract and amodal semantic system, that is accessed by the highest levels of the various perceptual modalities (“structural descriptions”), which include a complete perceptual specification of objects prior to their meaningful recognition. According to the above mentioned cognitive authors, there are no traces of the various sensory-motor modalities beyond the level of the corresponding “structural descriptions”, because the format of semantic representations is symbolic, abstract, amodal and propositional. On the contrary, the “empirical” line of thought has been endorsed in recent years by Allport (1985) and Jackendoff (1987). These authors challenged the model of an abstract and amodal conceptual/semantic system. They claimed that conceptual representations keep the stamp of the perceptual mechanisms through which they were formed and are stored in the same format in which they were constructed by the sensory-motor experience. Drawing in part on the latter cognitive models and in part on Hebb’s (1949) model of “cell assemblies”, Damasio (1989, 1990), proposed the dynamic construct of “higher-order convergence zones”, which assumes that concepts are retrieved by a process of recollection of modality-specific bits of memories, that are stored near the sensory portals and motor output sites of the system. Damasio’s (1989, 1990) construct of “higher-order convergence zones” was refined by Barsalou (1999, 2008), who added the similarity-in-topography (SIT) principle (Simmons and Barsalou, 2003), according to which the proximity of two conjunctive neurons in a convergence zone increases with the similarity of the features they conjoin. As a result, conjunctive neurons become topographically organized into local regions that represent properties and categories. From the viewpoint of the cognitive neurosciences, an important difference between the rationalist and the empiricist approach concerns the fact that these two lines of thought have different neurobiological implications concerning the anatomical correlates of conceptual representations. The rationalist approach, which assumes that conceptual categories are represented in an abstract and amodal format, has no reason to make predictions about the brain structures subsuming concepts in general or specific categories of knowledge. On the other hand, the empiricist approach, which assumes that conceptual categories result from the convergence in specific cortical areas of sensory-motor information that plays a leading role in the construction of each category, allows making predictions based on what is known about the brain structures processing this sensory-motor information.

In the following sections of this survey, I will first consider the neuropsychological data about category-specific semantic disorders on which all the theoretical models and the controversies among these models are based. Then I will identify the brain structures that process the sensory-motor information on which our knowledge of different categories could be based and their involvement in category-specific semantic disorders. My next step will be to analyze the arguments of inborn and experience-dependent models of categorical brain organization, including their interpretation of gender-related effects in category-specific semantic disorders and of the relationships between tool knowledge and the left ventral fronto-parietal areas. In this section I will discuss data which suggest that innate connectivity patterns mediate the integration of information critical for the organization of each category. Finally, I will survey experimental data supporting the importance of prior experience in the cortical representation of objects.

## Neuropsychological studies of category-specific disorders tend to support sensorimotor models of semantic knowledge

### The discovery of “category-specific” semantic disorders

The data on category-specific semantic disorders, on which all of the following theoretical models (and in particular the empiricists’ predictions) are based, can be traced back to a seminal series of papers in which Warrington et al. showed that the disruption of conceptual knowledge is not necessarily homogeneous across categories but is sometimes “category-specific” (Warrington and McCarthy, 1983, 1987; Warrington and Shallice, 1984). These category-specific semantic disorders usually affect the biological (“living”) more than the artifact (“non-living”) categories, but sometimes preferentially impair the artifact categories (see Saffran and Schwartz, 1994; Gainotti et al., 1995; Gainotti, 2000, 2005; Capitani et al., 2003 for reviews). Warrington et al. (Warrington and McCarthy, 1983, 1987; Warrington and Shallice, 1984) also noticed that in their patients the semantic disorders did not respect the boundaries between biological and artifact categories. For instance, the representation of “body parts” tended to be disrupted in association with the representation of artifact categories, whereas the representation of “musical instruments” tended to be disrupted in association with the representation of living items. This was explained as due to the fact that our knowledge of animals and musical knowledge is based on similar visual (shape) and acoustic (sound) information, whereas our knowledge of body parts and tools (or other artifacts) is mainly based on actions and somato-sensory information. More generally, these observations suggested that “category-specific semantic disorders” are not due to disruption of true “biological” and “artifact” categories, but are rather the by-product of a more basic dichotomy, concerning the differential weighting that visual-perceptual and functional attributes have in the representation of biological and, respectively, artifact categories. Warrington’s model (which has been called “the differential weighting hypothesis”) is based on the assumption that each conceptual representation derives from the convergence of different sensory, motor and verbal features, but that the weight of these features is different for different conceptual categories. For example, the distinction between a lion, a tiger and a leopard is mainly based on visual features, namely the plain, striped or spotted aspect of their skin. On the other hand, tools and other artifacts are distinguished by making reference to the actions they require and the functions they support and only marginally to their visual features. As the weight of the features that converge in a conceptual representation is related to the subject’s experience, this model implies that conceptual representations are experience-dependent. Furthermore, since Warrington et al. had maintained that our knowledge of biological entities is mainly based upon their perceptual features, whereas our knowledge of artifacts in mainly based upon their function, this model was called the Sensory/Functional Theory/SFT.

### Objections raised to the Sensory/Functional Theory and emergence of the “sensory-motor models of semantic knowledge”

Several objections were raised against the SFT by Caramazza et al. (e.g., Caramazza, 1998; Caramazza and Shelton, 1998; Capitani et al., 2003; Mahon and Caramazza, 2003; Caramazza and Mahon, 2006) and by other authors following a similar line of thought. The first objection was that there is no consistent correlation between conceptual impairment for living things and greater disruption of visual knowledge compared with functional knowledge (see Caramazza, 1998; Caramazza and Shelton, 1998; Mahon and Caramazza, 2001; Capitani et al., 2003 for discussion). There are, however, cases of intensively studied patients with category-specific semantic impairments for living things (e.g., Basso et al., 1988; Sartori and Job, 1988; Silveri and Gainotti, 1988; De Renzi and Lucchelli, 1994; Gainotti and Silveri, 1996; Rosazza et al., 2003) in whom the disproportionate impairment of the visual (i.e., compared with the functional) attributes predicted by the SFT, has been confirmed. On the other hand, the review by Capitani et al. (2003) raised methodological issues with some of those studies and the study by Rosazza et al. (2003) was challenged by Mahon and Caramazza (2004). In any case, the comparison between visual and functional knowledge of living beings is hindered by the need to define what is the meaning of a “functional” attribute when dealing with animals, in particular wild animals. More generally, “functional features” are a heterogeneous class of properties that includes actions accomplished with objects, notions about the objects’ use and verbally-mediated encyclopedic knowledge. This fact has been stressed, for instance, by Buxbaum et al. (2000), Buxbaum and Saffran (2002) and Boronat et al. (2005), who have distinguished, within functional knowledge, the function of an object from its manipulation, suggesting that since “manipulation” is related to a sensorimotor activity, it could be the component most tightly linked to the “differential weighting” hypothesis. Furthermore, the same authors showed that not only the properties denoted by the term “functional” are heterogeneous, but also those subsumed by the term “sensory” because different types of sensory data could have different weights in different kinds of semantic categories. Thus, visual perception could play a leading role in the mental representation of animals and somatosensory data in that of tools. These considerations led several authors (e.g., Gainotti et al., 1995; Chao et al., 1999; Gainotti, 2000, 2006; Martin et al., 2000; Martin and Chao, 2001; Martin, 2007; Barsalou, 2008) to suggest that the “sensory-functional theory” should be replaced with the “embodied cognition” theory (Barsalou, 2008) or with the “sensory-motor model of semantic knowledge”, (Gainotti et al., 1995; Chao et al., 1999; Gainotti, 2000, 2006; Martin et al., 2000; Martin and Chao, 2001; Martin, 2007). The latter takes into account various kinds of perceptual, functional, motor and verbally-coded properties, that can contribute to the construction of a conceptual representation. Other important models that have tried to explain the differences between biological and artifact categories on the basis of the different set of underlying features and of their interconnections are the correlated feature based (CFB) accounts (e.g., Tyler et al., 2000; Tyler and Moss, 2001; Randall et al., 2004; Bright et al., 2005, 2007; Taylor et al., 2009, 2011). These models underline the different levels of interconnections that exist between shared (perceptual and functional) attributes of living and non-living things and assume that this structural difference is more important than the differential weighting of perceptual and functional attributes in explaining category-specific semantic disorders. Although these models are interesting, they will not be taken into account here because they would not allow us to make clear predictions about the experience-dependent vs. innate models of conceptual representations.

### Objections to Warrington’s “differential weighting hypothesis”

The second objection raised by Caramazza et al. to the SFT is that the assumption of differential weighting of sensory and functional information in the representation of living things and artifacts is not systematically confirmed by studies conducted in normal subjects. In fact, Farah and McClelland (1991) and Caramazza and Shelton (1998) obtained conflicting results when they tried to assess the weight of different kinds of information in the representation of different conceptual categories in healthy subjects, by asking them to underline either visual or functional descriptors in dictionary definitions of living things or artifacts. Farah and McClelland (1991) found a much larger ratio of visual to functional attributes for living beings than for artifacts, whereas Caramazza and Shelton (1998) found only a non significant difference between these two domains of knowledge. These results were discrepant because in Farah and McClelland’s (1991) study a property was considered “functional” only if it described “what the item does or what it is for,” whereas in Caramazza and Shelton’s (1998) study all “non-sensorial” (namely functional, encyclopedic, etc.) descriptors were contrasted with the sensory properties. Feature lists were used by other authors (e.g., McRae and Cree, 2002; Cree and McRae, 2003; Vanoverberghe and Storms, 2003; Ventura et al., 2005; Zannino et al., 2006) to check the assumption of a differential weighting of sensory and functional information in the representation of living things and artifacts. These studies have generally provided an empirical support for the claim that sensory and functional features have different importance in the representation of living and non-living entities, but, as Hoffman and Lambon Ralph (2013) have rightly noted, feature lists may not give a complete picture of how knowledge is distributed amongst the various sensory-motor modalities available from our experience of the environment. Much more appropriate for testing the principles of the “sensory-motor model of conceptual knowledge” is a method proposed by Tranel et al. (1997b) and used by Gainotti et al. (2009, 2013a) and Hoffman and Lambon Ralph (2013). This method consists of asking normal subjects to evaluate (with Likert scales) the influence of different perceptual (visual, auditory, tactual, olfactory, and gustative) and motor activities, as well as encyclopedic information, in the mental representation of living and artifact categories. Using this procedure, all these authors showed: (a) that normal subjects consider the visual modality the main source of knowledge for all (biological and artifact) categories taken into account; and (b) that in biological categories the most important source of knowledge after vision is represented by other perceptual modalities, whereas in artifact categories it is represented by the actions performed with objects.

Apart from the major importance attributed to vision in the mental representation of every kind of concrete entity (which is not surprising if we consider that most of our knowledge of the world is acquired through this sensory modality) other perceptual data therefore prevailed in the representation of biological entities, whereas actions and somatosensory data prevailed in the representation of artifact categories. Taken together, all these data suggest that the greatest difference between living and artifact categories does not reside in the prominent role played by vision in the representation of living beings, and by functional features in the representation of artifacts, but in the interaction between visual data and other perceptual (auditory, olfactory, and gustatory) attributes in the case of living beings, and among visual data, action-related properties, and somato-sensory information, in the case of artifacts.

### The hypothesis of an “innate” categorical organization of conceptual knowledge

The third more general objection raised by Caramazza et al. (e.g., Caramazza, 1998; Caramazza and Shelton, 1998; Mahon and Caramazza, 2001, 2003; Capitani et al., 2003; Caramazza and Mahon, 2006) to the SFT (and other similar models) concerns the assumption that “category-specific semantic disorders” may be due to the different weight of different sources of knowledge in the construction of different categories, rather than to the disruption of true “biological” and “artifact” categories. Caramazza et al. labeled their interpretation of category-specific semantic disorders “the domains of knowledge’ hypothesis”; this model assumes the existence of an “innate” categorical organization of conceptual knowledge. More specifically, the “domains of knowledge” hypothesis’ posits that category-specific impairments for animals (potential predators), plant life (possible source of food), and artifacts reflect the disruption of innate brain networks, shaped by natural selection to support rapid identification of objects very relevant for survival. Thus, Caramazza et al. (e.g., Caramazza, 1998; Caramazza and Shelton, 1998; Capitani et al., 2003; Mahon and Caramazza, 2003; Caramazza and Mahon, 2006) questioned Warrington et al.’s (Warrington and McCarthy, 1983, 1987; Warrington and Shallice, 1984) statement that in their patients the semantic disorders did not respect the boundaries between biological and artifact categories, because the representation of “body parts” tended to be disrupted in association with that of artifacts, whereas the representation of “musical instruments” tended to be disrupted together with that of living items. These observations of Warrington et al. were, however, confirmed by several authors. For instance, Basso et al. (1988), Damasio (1990), Breedin et al. (1994), Farah et al. (1996), Silveri and Gainotti (1988) and Dixon et al. (2000) confirmed the association between living beings and musical instruments. Similarly, Basso et al. (1988), Hillis and Caramazza (1991), Goldenberg (1992), Hart and Gordon (1992), Breedin et al. (1994), De Renzi and Lucchelli (1994), Forde et al. (1997), Humphreys et al. (1997), Silveri and Gainotti (1988) and Kolinsky et al. (2002) confirmed the association between artifacts and body parts. However, Barbarotto et al. (2001) and Capitani et al. (2003) considered these results as spurious and due to the influence of unmatched “nuisance” variables, such as familiarity, age of acquisition and lexical frequency. On the other hand, this interpretation is at variance with data reported by Dixon et al. (2000), who confirmed the particular status of musical instruments, by using lists of living beings, artifacts and musical instruments that were well controlled for frequency, stimulus complexity and familiarity and by Masullo et al. (2012). Using a Semantic Knowledge Questionnaire (Laiacona et al., 1993), these authors studied a patient affected by a severe apperceptive visual agnosia, and found that the number of errors made on the musical instruments was similar to that obtained on the living categories of animals, fruits and vegetables and significantly higher of that made in the other artifact categories. This difference was still significant when familiarity, frequency of use and prototypicality of each stimulus entered into a logistic regression analysis. Furthermore, these clinical data were confirmed by experiments conducted with a neural network model (Gales et al., 2001), in which living things and musical instruments elicited greater recognition failures than the other categories. The discrepancy between the different accounts of the associations between biological entities and “musical instruments” given by the “sensory-motor model of semantic knowledge” and by the “domains of knowledge hypothesis”, is probably due to the fact that only a partial overlap exists between the sources of knowledge typical of these domains. Thus, visual and auditory attributes play an important role in the representation of both animals and musical instruments, but visual attributes are more important for animals and auditory features for musical instruments. Furthermore, motion is typical of animals, but not of musical instruments. Depending on the exact lesion location and accuracy in the control of nuisance variables, it is therefore possible that the association between animals and musical instruments may be due to joint disruption of their representations or to the influence of uncontrolled nuisance variables.

**In conclusion**, neuropsychological data are rather consistent with the “sensory-motor model of semantic knowledge”, because the main assumption of this model, namely the hypothesis that various kinds of perceptual, motor and verbally-coded properties may have differential weighting in the construction of different conceptual categories, is strongly supported by empirical data (e.g., Cree and McRae, 2003; Vigliocco et al., 2004; Gainotti et al., 2009, 2013a; Hoffman and Lambon Ralph, 2013). However, the “domains of knowledge hypothesis” raises important methodological objections to interpretations of the neuropsychological data based on the “differential weighting hypothesis”.

## The sensorimotor models of semantic knowledge allow explaining the cerebral correlates of biological and artifact categories

### Anatomical correlates of category-specific semantic disorders

Gainotti et al. (1995), Martin et al. (1996) and Gainotti (2000) were the first authors to suggest that the anatomical locus of lesion in category-specific semantic disorders might be informative about the nature of the underlying cognitive impairment. The title of Gainotti’s (2000) paper (“What the locus of brain lesion tells us about the nature of the cognitive defect underlying category-specific disorders”) explicitly stressed this point, which had been hitherto neglected because previous (cognitively oriented) authors (e.g., Warrington and Shallice, 1984; Hart et al., 1985; Warrington and McCarthy, 1987; Sartori and Job, 1988), who had reported the anatomical locus of lesions in their patients, had not proposed a general interpretation of the relationships between clinical and anatomical data. Gainotti (2000) argued that, if distinctive sources of knowledge play a critical role in the construction of different semantic categories, then the brain structures disrupted in a given type of category-specific semantic disorder should correspond to the areas of convergences of the sensory-motor information which has played a major role in the construction of that category. More specifically, the anterior parts of the temporal lobes (where the ventral stream of visual processing converges with auditory, olfactory and gustatory inputs) should play a critical role in the representation of biological entities; and the fronto-temporo-parietal, sensory-motor cortices (where the dorsal stream of visual processing converges with body-related and action-oriented structures) should play a major role in the representation of artifacts. Furthermore, a different degree of lateralization of the brain representation of biological entities and artifacts should be predicted, because the main sources of knowledge about living beings (namely visual and other perceptual inputs) are bilaterally represented, whereas (in right-handed subjects) the action oriented structures, which provide an important source of knowledge about artifacts, are mainly represented in the left hemisphere, which controls the movements of the right side of the body. Both of these predictions are substantiated by a number of anatomo-clinical and neuroimaging studies. Several reviews of the anatomical correlates of category-specific semantic disorders (e.g., Saffran and Schwartz, 1994; Gainotti et al., 1995; Damasio et al., 1996; Tranel et al., 1997a; Gainotti, 2000, 2005, 2006; Capitani et al., 2003), have, indeed, confirmed the critical role played by lesions of the anterior parts of the temporal lobes in semantic disorders for biological entities. These reviews showed that brain structures located in the rostral parts of the ventral stream of visual processing (such as the infero-temporal (IT) cortices) or integrating highly processed visual data with other sensory modalities (such as the perirhinal and entorhinal cortices) are usually disrupted in patients with category-specific semantic disorders for living things. Data consistent with these views were also obtained by Strauss et al. (2000) and by Luckhurst and Lloyd-Jones (2001), who showed that temporal lobectomy patients are disproportionately more impaired in naming living than nonliving entities.

### Results of functional neuroimaging investigations

Data in agreement with this model were also obtained in a series of neuroimaging studies by Grabowski et al. (2001), Devlin et al. (2002), Tyler et al. (2004), Bright et al. (2005) and Moss et al. (2005). These authors showed that the human perirhinal cortex and neighboring anterior temporal structures provide the neural infrastructure for living categories. For instance, Devlin et al. (2002) entered data from seven PET studies into a single multifactorial design which crossed category (living vs. man-made) with a range of tasks and found that living things activated medial aspects of the anterior temporal poles bilaterally and that tools activated a left posterior middle temporal region. On the other hand, Bright et al. (2005) reviewed recent neuropsychological and neuroimaging studies and found that the human perirhinal cortex and contiguous anteromedial temporal structures provide the neural infrastructure for making fine-grained discriminations among objects. This suggests that damage within the perirhinal cortex may underlie the emergence of category-specific semantic deficits for living things.

If we pass from living beings to artifacts, we see that lesions of a network involving the infero-lateral part of the left frontal lobe, the left inferior parietal lobe and the left middle temporal gyrus, where different components of action schemata are represented (see Saygin et al., 2004), provoke prevalent impairment for tools and other man-made artifacts, whose knowledge is mainly based upon active manipulation and physical contact with objects. This claim is not only supported by the results of Gainotti’s (2000) systematic review of the anatomical correlates of category-specific semantic disorders, but also by other more recent reviews (e.g., Capitani et al., 2003; Kellenbach et al., 2003; Gainotti, 2005, 2013; Buxbaum and Kalénine, 2010; Campanella et al., 2010).

**Figure 1:**
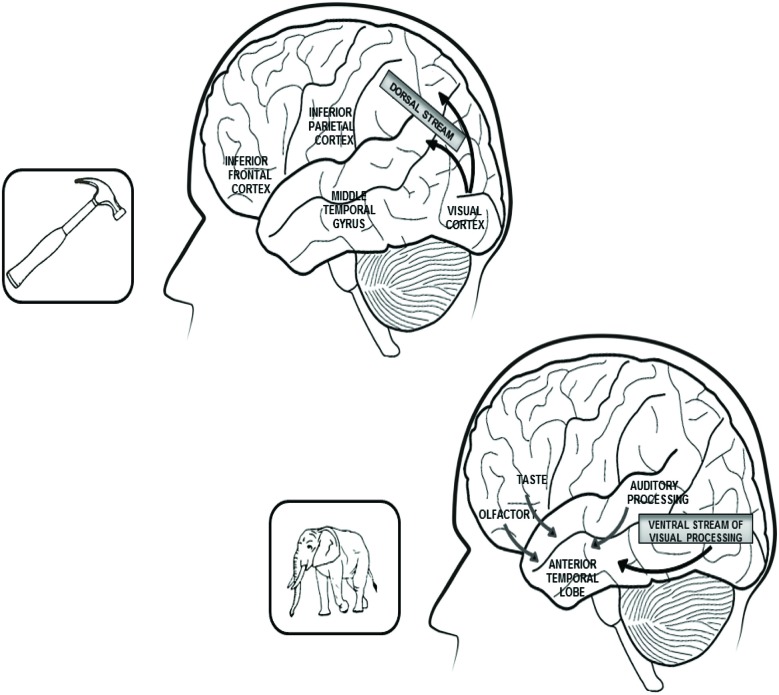
**In the upper part of the figure are reported the left hemisphere brain structures that play a critical role in the representation of tools and other artifacts**. Within this network, the inferior frontal cortex subsumes action-related features, the inferior parietal cortex is connected with somato-sensory information and the middle temporal gyrus processes visual information, coming through the dorsal stream of visual processing and concerning the objects’ movements. In the lower part of the figure are reported the connections between the anterior temporal lobes, which bilaterally subsume the representations of living beings and the sources of knowledge that mainly contribute to the construction of these representations: visual and auditory features for the category of animals and visual, gustatory and olfactory features for the plant-life categories (flowers, fruits and vegetables). Both right and left anterior temporal lobes are equally involved in the representation of living beings because the perceptual sources of knowledge on which these categories are based are bilaterally represented. By contrast, the network subsuming tools and artifacts knowledge is lateralized to the left hemisphere because the action-related and somato-sensory information on which these categories are based come (in right-handed subjects) from the right side of the body.

**In conclusion**, results of anatomo-clinical and of functional neuroimaging investigations confirm that distinctive sources of knowledge have a critical role in the construction of different semantic categories and that the brain structures disrupted in a given type of category-specific semantic disorder (or activated in neuroimaging experiments using items from the same category) overlap with areas of convergence of sensory-motor information which has a major role in the construction of that category. The main results of anatomo-clinical and of functional neuroimaging investigations on the cerebral correlates of biological and artifact categories are reported in Figure 1.

## Inborn and experience-dependent models of categorical brain organization and the interpretation of gender-related effects

### The domains of knowledge’ hypothesis

In previous sections of this review, we saw that the “embodied cognition” theory (Barsalou, 2008) and the “sensory-motor model of semantic knowledge”, (Gainotti, 2000, 2006; Martin and Chao, 2001; Martin, 2007) can account for the neuropsychological aspects of category-specific semantic disorders (even if the evidence and the arguments that have been made against those theories cannot be ignored) and allow for correct predictions about the brain structures subsuming specific categories of knowledge. Nevertheless, these models are at variance with the “domains of knowledge’ hypothesis”, proposed by Caramazza et al. (Caramazza, 1998; Caramazza and Shelton, 1998; Capitani et al., 2003; Caramazza and Mahon, 2006), which assumes the existence of an “innate” categorical organization of conceptual knowledge, and posits that category-specific impairments for animals (potential predators), plant life (possible source of food), and artifacts reflect the disruption of innate brain networks, which are shaped by natural selection to support rapid identification of objects that are very important for survival. The distinction within “living beings” between category-specific disorders for animals and plant life is certainly correct. It is, indeed, supported by at least three sources of evidence: (a) results of hierarchical cluster analyses used in feature listing studies (e.g., Cree and McRae, 2003; Vigliocco et al., 2004 ) or in investigations conducted using Likert scales (e.g., Gainotti et al., 2009, 2013a; Hoffman and Lambon Ralph, 2013), which have documented a tripartite organization of knowledge (with three major clusters corresponding to animals, fruits and vegetables and artifacts); (b) single case studies of patients showing a selective impairment for animals (e.g., Damasio et al., 1990; Hart and Gordon, 1992; De Renzi and Lucchelli, 1994; Caramazza and Shelton, 1998; Blundo et al., 2006) or for fruits and vegetables (e.g., Hart et al., 1985; Hanley et al., 1989; Farah and Wallace, 1992; Goldenberg, 1992; Forde et al., 1997; Kensinger et al., 2003; Samson and Pillon, 2003; Siri et al., 2003); and (c) results of a group study conducted by Capitani et al. (2009), which showed that the anatomical locus of lesion is not exactly the same in patients with selective disorders for animals and plant life stimuli.

### The interpretation of gender-related effects

The claim that the difference between animals and plant life categories is inborn and shaped by natural selection was, however, questioned by Gainotti (2005, 2010). This author’s reviews showed that only the distinction between biological and artifact categories reflects an anatomically-based categorical organization and that the discrepancy (within the living entities) between animals and plant life categories is mainly due to gender and social roles related familiarity factors. In fact, Gainotti’s (2005, 2010) reviews showed that in patients with category-specific semantic impairments for living beings men were systematically more impaired with plant life categories and women were usually more impaired with animals. The presence of a gender effect was not unexpected, because an interaction between gender and familiarity with different kinds of living and non-living categories had already been documented by several authors. In a normative study conducted by McKenna and Parry (1994) and in later investigations conducted in normal subjects by Capitani et al. (1999), Laws (1999, 2000, 2004), Albanese et al. (2000), Barbarotto et al. (2002) and Funnell et al. (2006), men were usually more familiar with artifacts and women with living things. Furthermore, a detailed analysis of data gathered in normal subjects suggested a further gender by category interaction even within biological categories, because men were more proficient with animals and women with fruits and vegetables (McKenna and Parry, 1994; Albanese et al., 2000; Laws, 2004; Barbarotto et al., 2008; Cameron et al., 2008). What was surprising, however, was the size of the gender effect. In fact, in Gainotti’s (2005) review 18 out of the 19 patients who showed a selective impairment for fruits and vegetables were men, whereas 7 out of the 9 patients who showed a prevalent impairment for animals were women. Gainotti (2005) and Marra et al. (2007) explained these gender effects on the basis of the greater familiarity that men might have with tools and with certain kinds of animals (probably because they are more involved in hunting activities) and women for fruits and vegetables (probably because of their cooking activities).

An attempt to reconcile these gender effects with inborn models of categorical brain organization was proposed by Laws (2000, 2004) and developed by Laiacona et al. (2006). According to Laws (2000, 2004), greater development of brain circuits dealing with tools and animals in men and with fruits and vegetables in women may have been produced by the main subsistence activities of men (hunting) and women (gathering). Refining this line of thought, Laiacona et al. (2006), proposed that the evolutionary pressures which prompted the development of different brain networks dedicated to animals, plant life and tools might also have provided each gender with more efficient cognitive representations of their main working and foraging targets (i.e., tools and animals for men and fruits and vegetables for women). This inborn account of gender effects, based on evolutionary pressures is, however, at variance with the fact that gender effects do not respect the boundaries between artifacts, animals and plant life categories. Several authors (e.g., Albanese et al., 2000; Moreno-Martinez et al., 2008; Gainotti et al., 2013b) have shown that in the artifact categories, men fare better with tools and women with furniture and kitchen utensils. An objection that could be raised to the “greater familiarity hypothesis” is that, given that in developed countries men no longer hunt and women share their cooking activities with men, gender-related categorical effects should have disappeared by now. This, however, is exactly what was found by Moreno-Martinez et al. (2008) and by Gainotti et al. (2010, 2013b). No difference was observed by Moreno-Martinez et al. (2008) or by Gainotti et al. (2010) in any of the categories considered in their studies when young males and females (who belonged to a generation in which the traditional social roles have almost completely disappeared) were taken into account. The situation changed, however, when elderly subjects were investigated. In Moreno-Martinez et al.’s (2008) study, elderly females obtained better results with flowers, vegetables and kitchen utensils, whereas elderly males obtained better scores with musical instruments. In Gainotti et al.’s (2013b) study elderly men showed a greater familiarity for animals and women for flowers. Furthermore the suggestion advanced by Marra et al. (2007) that the higher male familiarity with animals might derive from their hunting activities was confirmed by Scotti et al. (2010). Then, they subdivided them into various categories and showed that males were more familiar with hunted animals.

**In conclusion**, data showing that gender effects do not respect the boundaries between artifacts, animals and plant life categories are clearly more consistent with an experience-dependent interpretation of gender-related asymmetries (Gainotti, 2005, 2010; Marra et al., 2007) than with the assumption that evolutionary pressures may have provided each gender with the most efficient cognitive representations of their main working and foraging targets (Laws, 2000, 2004; Laiacona et al., 2006).

## Inborn and experience-dependent interpretations of the relationships between tool knowledge and the left ventral fronto-parietal areas

In Section “Neuropsychological Studies of Category-Specific Disorders Tend to Support Sensorimotor Models of Semantic Knowledge” of this review, regarding the functional knowledge of objects, I pointed out that Buxbaum et al. (2000) and Buxbaum and Saffran (2002) distinguished between knowledge of the function of objects and that of their manipulation, suggesting that “manipulation” (being related to a sensorimotor activity), might be the component more tightly linked to the “differential weighting” hypothesis. Kellenbach et al. (2003) and Boronat et al. (2005) confirmed this hypothesis in two functional magnetic resonance imaging (fMRI) experiments. They asked normal subjects to make judgments about actions and functions associated with manipulable and non-manipulable objects. Both studies showed that the left inferior frontal and parietal areas responded more strongly to actions (vs. functions) and to manipulable (vs. non-manipulable) objects. These results confirmed that brain regions specialized for sensory-motor functions play a critical role in the representation of tools and other manmade objects.

### The “embodied cognition hypothesis” and the relationship between manipulation, tool knowledge and left ventral fronto-parietal areas

Some theoretical models have been advanced to explain the relationship between manipulation, tool knowledge and left ventral fronto-parietal areas. One of these models is based on a strong version of the “embodied cognition hypothesis” (Barsalou, 1999, 2008; Gallese and Lakoff, 2005) and maintains that the conceptual processing of tools necessarily involves the retrieval or simulation of the movements associated with tool usage. According to this view, motor programs are run during object recognition and are necessary to ground the conceptual knowledge of objects. One prediction that can be made on the basis of this hypothesis is that loss or impairment of motor programs concerning the use of tools should be associated with disruption of the corresponding conceptual knowledge. But data obtained in brain-damaged patients have provided results that were inconsistent with this strong version of the “embodied cognition theory”. Thus, research conducted in patients with apraxia, whose performance is impaired when they imitate observed actions, by using objects or pantomiming their use from visual presentation, has shown that the ability to use objects may be much more impaired than naming them or knowing their function (Buxbaum et al., 2000; Buxbaum and Saffran, 2002; Rosci et al., 2003; Negri et al., 2007). Furthermore, results of two recent studies are at variance with the strong version of the “embodied cognition theory”. In one of these studies, Arévalo et al. (2012) presented left hemisphere stroke patients with pictures and words representing objects and actions typically associated with use of the hand, mouth and foot. They correlated results obtained on these tasks with data obtained from voxel-based lesion-symptom mapping analyses, but found no support for a correlation between body parts involved in the use of objects and somatotopically organized locus of damage. In another single case study, Garcea et al. (2013) reported the detailed investigation of a patient with a large left hemisphere lesion whose object knowledge was relatively spared in spite of a severe motor (action production) defect and impaired conceptual knowledge of actions. Taken together, the few studies that have used lesion data to test predictions deriving from a strong version of the “embodied cognition theory” provided data inconsistent with this theory.

### The innately determined connectivity patterns suggested by “the distributed domain-specific hypothesis”

Other theoretical models acknowledge that motor programs associated with tool use have an important role in the construction of tool representation, but deny that a necessary and sufficient relationship exists between the re-enactment of these sensory-motor processes and tool knowledge.

These models are the “domains of knowledge” hypothesis, (Caramazza, 1998; Caramazza and Shelton, 1998; Mahon and Caramazza, 2001, 2003; Caramazza and Mahon, 2006) and the “sensory-motor model of semantic knowledge”, (Gainotti et al., 1995; Chao et al., 1999; Gainotti, 2000, 2006; Martin and Chao, 2001; Martin, 2007), which have already been discussed in previous sections of the present review. The basic difference between these two models is that the domains of knowledge hypothesis is an innatist model and the sensory-motor model of conceptual knowledge maintains that categorical brain organization is experience-dependent. In fact, the domains of knowledge hypothesis’, which in its first formulation did not consider as relevant the anatomical correlates of brain categorical organization, after the publication of data obtained by Gainotti (2000, 2005) in patients with category-specific semantic disorders and by Kellenbach et al. (2003), Tyler et al. (2004), Bright et al. (2005) and Moss et al. (2005) in functional neuroimaging experiments, acknowledged that the neural substrate of each domain of knowledge consists of a network where the information most relevant for that category converges. However, this model, called “the distributed domain-specific hypothesis” by Mahon and Caramazza (2009, 2011), argues that innately determined connectivity patterns mediate the integration of information critical for the organization of each domain of knowledge; by contrast, the sensory-motor model of conceptual knowledge holds that each category results from the convergence of different sources of knowledge whose organization is experience-dependent. According to the distributed domain-specific hypothesis (Mahon and Caramazza, 2009, 2011), a domain-specific neural system is a network of brain structures in which each region processes a different type of sensory, motor, affective or conceptual information about the same category of objects. Furthermore, the computations that must be performed on items in the same category are sufficiently specific to merit a specialized process. For instance, there is a strong need to integrate motor-relevant information with visual information for tools and other artifacts; this need is less strong for animals and faces. Similarly, there is a strong need to integrate affective information, biological motion processing and visual form information for animals and conspecifics; this need is less strong for tools and other artifacts. Thus, supporters of the distributed domain-specific hypothesis propose that specialization for faces in the lateral fusiform area of the ventral visual stream occurs because this region of the brain is connected with the amygdale and the superior temporal sulcus, which are important for the extraction of socially relevant information. By contrast, specialization for tools and manipulable objects is driven by the connectivity between the inferior frontal and parietal cortex, which subserve object manipulation and regions of the medial fusiform gyrus, that are involved in the visual processing of tools.

### Data suggesting the innate nature of the categorical brain organization and objections raised to the hypothesis that innate connectivity patterns may underlie categorical organization

Strong empirical data supporting the innate nature of these patterns of connectivity come from work indicating that congenitally blind subjects show activation for words (presented in Braille) in the same regions of the ventral stream that are activated by visually presented words in sighted individuals (Buchel et al., 1998). Furthermore, Mahon et al. (2009) showed that the same medial-to-lateral bias in category preferences for artifacts vs. animals, which is present in the ventral surface of the temporo-occipital cortex in sighted individuals (Chao et al., 1999; Noppeney et al., 2006; Mahon et al., 2007), is also present in congenitally blind subjects. Mahon et al. (2009) suggested that, if visual experience is unnecessary for the emergence of category-specificity in the ventral stream, innate connectivity between regions of the ventral stream and other regions of the brain could drive category-specificity. Some objections have, however, been raised to this claim. First, several authors (e.g., Downing et al., 2006; Mechelli et al., 2006; Chouinard et al., 2008; Pourtois et al., 2009; Cate et al., 2011; Taylor and Downing, 2011) have challenged the specificity of the medial-to-lateral bias in category preferences. Second, according to Kiefer and Pulvermüller (2012) these data do not necessarily suggest that innate connections underlyie the organization of object knowledge, because the brain of congenitally blind patients is subject to many plastic changes in which visual areas are activated by tactile (Sadato et al., 1996; Röder et al., 1997) and auditory (Klinge et al., 2010; Collignon et al., 2011) information. It is, therefore, possible that tactile and auditory processing of biological and artifact stimuli recruits “visual” areas in the course of cortical reorganization and leads to the extraction of category-specific object properties, similarly to what occurs in visual exploration. Authors who argue that innately determined connectivity patterns mediate the integration of information critical for the organization of each domain of knowledge do not deny that the brains of blind subjects are different from the brains of sighted participants in important ways nor that “visual” regions in the blind process tactile information (e.g., Mahon et al., 2009; Amedi et al., 2010). In fact, they hold that the relevant finding for constraining models is not the format of the information that is represented but rather its organization. On the other hand, it can be objected that, to devise a task that could be performed by both sighted and blind individuals, Mahon et al. (2009) asked participants to make size judgments about stimuli that were presented as auditory words. These size-judgment tasks can be performed on the basis of both visual and somato-sensory kinesthetic information. Now, if we look at the original data of Mahon et al. (2009), we see that only activation concerning the artifacts, which emerged in the mesial parts of the ventral surface of the occipital-temporal cortex, was strong and spatially extensive; by contrast, activation concerning living beings (in the lateral parts of the same cortical areas) was weak and limited to small spots. This may have been because congenitally blind participants have disproportionately more somato-sensory experience, which is much more relevant for processing the shapes of nonliving (e.g., a fork, a car) than living things.

Other objections to the innatist model of the distributed domain-specific hypothesis come from results, reported by Baker et al. (2007) in humans and by Srihasam et al. (2012) in monkeys. Baker et al. (2007) showed that category-specific regions in the ventral visual pathway, such as the “visual word form area” (VWFA), can be created through visual experience, without a strong genetic predisposition for that specific selectivity. These authors used high resolution fMRI to scan both Hebrew and non-Hebrew readers while they viewed English words, Hebrew words, Chinese characters and line drawings and found a small region in the left hemisphere fusiform gyrus, that selectively responded to letter strings. This region responded more strongly to Hebrew words in Hebrew readers than in non- Hebrew readers, indicating that the striking selectivity of this region for one class of stimuli originates from extensive experience with that stimulus class. Srihasam et al. (2012) conducted experimental studies in monkeys to evaluate whether the existence of dedicated cortical domains necessarily means that the corresponding abilities are innate, or whether these domains can be formed or refined by interactions between genetic programs and common early experience. They showed that intensive early, but not late, experience caused the formation of category-selective regions in the macaque temporal lobe for stimuli (i.e., letters and numbers) never encountered naturally by monkeys. Their explanation of these results was that thanks to a self-organizing activity-dependent Hebbian mechanism, intensive early experience drives the segregation of category-specific domains in the cortical areas of the IT cortex. It must be acknowledged, on the other hand, that the positions of Mahon and Caramazza (2009, 2011) were supported by a set of recent fMRI data from blind individuals obtained by Striem-Amit et al. (2012). These authors, taught congenitally blind adults to read and recognize complex images using sounds that topographically represented images (“soundscapes”). The blind subjects selectively activated the VWFA during the processing of letter soundscapes relative to textures or visually complex object categories. Therefore, similar to the blind subjects tested by Mahon and Caramazza, the VWFA of their blind participants showed category selectivity regardless of the input sensory modality, visual experience, and long-term familiarity or expertise with the script. Striem-Amit et al. (2012) thus concluded that, although the VWFA is located in classical “visual” regions of the brain, the sensory format of the stimulus might already be “abstracted away” when the information arrives in the fusiform gyrus, allowing the same information to be processed by that particular structure, even when it is channeled through other modalities.

In any case, the recruitment of occipital regions in the congenitally blind, the ability of the brain to reorganize itself due to experience and the observation of specialized cognitive modules in the occipital cortex of congenitally blind, similar to those observed in the sighted, are still the subject of intense debates (see Held et al., 2011; Ricciardi and Pietrini, 2011; Struiksma et al., 2011; Bedny et al., 2012; Collignon et al., 2013; Peelen et al., 2014 for different viewpoints on these subjects).

**In conclusion**, data indicating that the medial-to-lateral bias in category preferences for artifacts vs. animals, which is present in the visual “ventral stream” of sighted individuals, is also present in congenitally blind subjects certainly suggest that, if visual experience is not necessary for the emergence of this categorical neural organization, innate patterns of connectivity must mediate the integration of information critical for the organization of each category. These suggestions must, however, be taken with caution, because an interaction has been hypothesized in critical periods of development between an inborn general brain organization (allowing the connection between the main sensory-motor, cognitive, executive and affective brain structures) and an experience-dependent actualization or reshaping of this basic brain design. Furthermore, data obtained in humans and in monkeys suggest that, thanks to a self-organizing activity-dependent Hebbian mechanism, intensive early experience drives the segregation of category-specific domains within cortical areas in the IT cortex.

## The importance of prior experience in the cortical representation of objects

Several recent investigations tried to assess the importance of prior perceptual and motor experience in the cortical representation of previously familiar or unknown objects, whose knowledge had been learned through intensive training. These lines of research will be analyzed in some detail. First, the results of investigations that studied the influence of experience with previously familiar objects or tasks will be reported and then the results of experimental studies that evaluated the influence of training on previously unfamiliar material.

### The influence of experience with previously familiar objects

The first line of research was pursued by Hoenig et al. (2011) in the auditory domain and by Yee et al. (2013) in the motor domain. Hoenig et al. (2011) started from the premise that professional musicians constitute a very good model for understanding experience-dependent plasticity in the human brain and investigated whether this neuroplasticity might extend beyond basic perceptual and motor functions and shape the semantic representation of musical instruments. Using fMRI, they showed that in musicians (but not in musical laypersons) conceptual processing of visually presented musical instruments activates the auditory association cortex encompassing the right posterior superior temporal gyrus, which is also recruited in the auditory perception of real sounds. Therefore, experience-driven neuroplasticity in musicians is not confined to alterations of perceptual and motor maps but also leads to the establishment of higher-level semantic representations for musical instruments. Yee et al. (2013) assessed the extent to which previous motor experience is part of an object’s representation. They showed: (a) that when the hands are engaged in a task involving movements that are incompatible with those used to interact with frequently manipulated objects, it is more difficult to make verbal judgements about those objects; and (b) that the amount of manual experience with the objects determines the amount of interference.

### The influence of training on previously unfamiliar material

The second research strategy, which started from the fact that the history of previous sensory-motor experience with familiar objects cannot be controlled, was based on the use of previously unfamiliar material and the administration of different types of extensive training with these objects. This line of research was followed by James and Gauthier (2003), Creem-Regehr et al. (2007), Kiefer et al. (2007), Weisberg et al. (2007) and Bellebaum et al. (2013).

James and Gauthier (2003) asked participants to learn associations between novel objects (“greebles”) and verbal labels of object features referring to a given modality (auditory and object motion). In a sequential matching task at test, the authors found stronger activity to objects associated with auditory words (“buzzes”) in the superior temporal gyrus, which responds to sounds in general, and stronger activity for objects associated with motion words (“hops”) in the superior posterior temporal sulcus, which is sensitive to motion processing. Similarly, Kiefer et al. (2007) assessed the plasticity of conceptual representations by training subjects with novel objects under different training conditions. In one class of stimuli object categorization was based on a detail feature, affording a particular action. During training, participants were asked either to make an action pantomime toward the detail feature or simply to pay attention to it by pointing to it with their index finger. In a categorization task at test, the neural correlates of the acquired conceptual representations were assessed. In the pantomime group, in which a meaningful action was performed towards the object during training, early activation was found in the frontal areas; but in the pointing training group, in which the action during training was not related to the object, these effects were absent. These results show that action information contributes to conceptual processing according to the specific learning experience, and suggest that conceptual representations are established by the learning-based formation of cell assemblies in different cortical areas. On the other hand, Creem-Regehr et al. (2007) used fMRI to investigate the influence of action knowledge associated with viewing, grasping, and using novel graspable objects. Participants were trained to perform complex actions associated with novel objects (“tools”) and had experience manipulating other visually similar novel objects (“shapes”). During scanning participants viewed, imagined grasping, and imagined using the objects. The greatest differences between “tools” and “shapes” were found in the “using” condition, in which greater effect sizes were observed for tools vs. shapes in the left inferior parietal lobule (IPL), pre-supplementary motor cortex (pre-SMA) and, marginally, in the left ventral premotor cortex (VPM). These results suggest that representations of tools are constructed on the basis of complex action schemata, which recruit processes related to graspability, action plans and use of objects. Similar conclusions were reached by Weisberg et al. (2007), who assessed the learning of tool-like functions for novel objects in an fMRI experiment in which subjects had to visually match pictures of novel objects before and after extensive training in the use of these objects to perform specific tool-like tasks. Compared with a pre-training baseline, activity increased after training in brain regions associated with motion (left middle temporal gyrus) and with manipulation (left intraparietal sulcus and premotor area) of tools and other manipulable objects. Furthermore, activations in the ventral temporo-occipital cortex became more focal after training. Specifically, although activity was widespread in the fusiform gyrus prior to training, activity, after training it was markedly increased in the medial portion of the fusiform gyrus, which is associated with identifying common tools, and was markedly reduced in the more lateral parts of the fusiform gyrus (i.e., in regions preferring animate objects like animals and faces).

Finally, Bellebaum et al. (2013) used fMRI to study the impact of different types of object-related sensorimotor experiences on the neural representations of novel objects, by contrasting manipulation training (MTO) with visual training (VTO) and absence of training (NTO). The post-training activity in the left inferior/middle frontal gyrus and the left posterior IPL was higher for MTO than for VTO and NTO, suggesting that manipulation experience leads to greater activity specifically in regions of the fronto-parietal cortex.

**In conclusion**, these studies assessing the importance of perceptual and motor experience in the cortical representation of previously familiar or of previously unknown objects, confirm the importance of previous experience in the learning-based formation of cortical cell assemblies subsuming the cortical representation of concepts. The implication of these results is that, if it is shown that experience shapes brain organization then there is evidence against inborn models. The problem with this argument is that inborn models do not deny that experience shapes brain organization i.e., that all of the content that an individual represents comes from experience. The question is whether the basic organization by category is something that depends only on experience or also on endogenous constraints.

## General discussion and tentative concluding remarks

If we try to summarize the data gathered in the present review, which are synthesized in Table 1 (by integrating the results of the different lines of research with some of the more important papers illustrating these lines) we can say that there are two main reasons why it is difficult to choose between the experience-dependent and the inborn models of conceptual representations.

**Table 1:** **Overview of some of the most relevant papers that have supported either the innate or the experience-dependent models in the various lines of research surveyed in the present review**.

Authors	Method	Results	Conclusions
**1. Neuropsychological features of patients with category-specific semantic disorders**			
Experience-dependent models maintain that these disorders are only apparently category-specific, but do not respect the boundaries between various categories and are due to the differential weight that different attributes have in the construction of different categories.			
Warrington and Shallice (1984)	Behavioral study of four patients with HSE	Category-specific semantic disorders for living beings and musical instruments are reported	The defects are not due to true categorical impairments but to the differential weight that different attributes have in the construction of various categories. Defects, indeed, do not respect the boundaries between different categories.
Innate models maintain that these patients can have disturbances restricted to a specific semantic category, that cannot be explained on the basis of perceptual, functional or encyclopedic disorders.			
Caramazza and Shelton (1998)	Review of the literature and results of a new case of category-specific semantic deficit	The selective damage of knowledge about animals is due to truly categorical effects.	The (innate) domain-specific knowledge framework provides a better account of category-specific deficits than the sensory/functional dichotomy theory proposed by Warrington and Shallice.
**2. Relationships between the anatomical locus of lesions in category-specific semantic disorders and the processing of information playing a critical role in the construction of these categories**.			
Experience-dependent models have predicted that, if distinctive sources of knowledge play a critical role in the construction of different semantic categories, then the brain structures disrupted in a given type of category-specific semantic disorder should overlap with the areas of convergence of sensory-motor information, which has a major role in the construction of that category.			
Gainotti (2000)	Review of the neuroanatomical correlates of living and non-living disorders.	Bilateral lesions of the anterior temporal lobes (ATL) were found in patients with living disorders. Patients with non-living disorders had left fronto- parietal (F-P) damage	In the ATLs the ventral stream of visual processing converges with auditory, olfactory and gustatory inputs, typical of living beings. In the left F-P areas the dorsal stream of visual processing converges with body- and action-related inputs coming from the right side of the body
Innate models have made no prediction on this point, because they were interested in the cognitive aspects and not in the anatomical correlates of category-specific semantic disorders.			
Capitani et al. (2003)	Review of clinical evidence in the field of semantic category-specific deficits	The review showed that category-specific defects concern animals, plant life and artifacts. Anatomical data were also reported	The authors argued against the sensory/functional theory and claimed that the boundaries between different domains of knowledge are respected, but did not discuss the cognitive implications of the neuroanatomical data.
**3. Gender-related categorical effects showing a prevalence of tools and animal knowledge in men and of fruits and vegetable knowledge in women**.			
Experience-dependent models have explained these gender effects on the basis of the greater familiarity that men might have with tools and animals (because of hunting activities) and women might have for fruits and vegetables (because of their cooking activities).			
Innate models have proposed that the evolutionary pressures which prompted the development of different brain networks dedicated to animals, plant life and tools might also have provided each gender with more efficient cognitive representations of their main working and foraging targets			
Moreno-Martinez et al. (2008)	Semantic fluency administered to old and young men and women	No gender effect was found in young people. Old women obtained better results with flowers and kitchen utensils, old men with musical Instruments	The fact that gender effects do not respect, in old people, the boundaries between artifact, animal and plant life categories is at variance with the innate and supports the “familiarity” hypothesis (see text for detailed account).
**4. Investigations which have tried to assess the importance of prior perceptual and motor experience in the cortical representation of previously familiar or unknown objects**			
Evidence: investigations which have studied the influence of experience with previously familiar objects and have evaluated the influence of training on previously unfamiliar material have consistently confirmed the importance of previous experience in the learning-based formation of cortical cell assemblies, that subsume the cortical representation of concepts.			
**5. Similar organization of semantic categories in the ventral surface of the temporo-occipital cortex in sighted individuals and in congenitally blind subjects**.			
Mahon et al. (2009)	asked sighted and blind individuals to judge the size of stimuli that were presented as auditory words	They showed that the same medial-to-lateral bias in preference for artifacts vs. animals is present in the ventral surface of the temporo-occipital cortex of sighted individuals and of congenitally blind subjects	They suggested that, if visual experience is unnecessary for the emergence of category-specificity in the ventral stream, an innate connectivity between this and other regions of the brain could drive category-specificity.

The first reason is that both models acknowledge that experience shapes brain organization and that all of the content an individual represents comes from experience. The question is, therefore, whether the basic organization by category is something that depends only on experience or also on endogenous constraints. The second reason is that some of the empirical data gathered in this survey stress the importance of experience-related factors, other data suggest the existence of endogenous constraints and still other data do not clearly support either of these mechanisms. In the data stressing the importance of experience-related factors, I would mention: (a) the sizeable gender effects observed by Gainotti (2005) in reviewing case reports of category-specific semantic impairment; and (b) results of experimental studies which have shown that perceptual and motor experience play a critical role in the cortical representation of previously familiar or unknown objects.

Gender-related categorical effects were explained by Gainotti (2005) as due to men’s greater familiarity with tools and with certain kinds of animals (because they are more involved in manual and hunting activities) and women’s greater familiarity with fruits and vegetables (probably as a result of their cooking activities). An inborn account of these gender effects, proposed by Laws (2000, 2004) and by Laiacona et al. (2006) is at variance with the fact that these effects do not respect the boundaries between artifacts, animals and plant life categories. The observation that within the artifact categories men fare better with tools and women with “furniture” and “kitchen utensils” (Albanese et al., 2000; Moreno-Martinez et al., 2008; Gainotti et al., 2013b) is consistent with an “experience-dependent” interpretation of gender-related asymmetries, but not with the assumption that evolutionary pressures may have provided each gender with the most efficient cognitive representations for their main work and foraging targets.

As for the importance of perceptual and motor experience in the cortical representation of previously familiar or unknown objects, investigations which have studied the influence of experience with previously familiar objects (Hoenig et al., 2011; Yee et al., 2013) and results of experimental studies which have evaluated the influence of training on previously unfamiliar material (James and Gauthier, 2003; Creem-Regehr et al., 2007; Kiefer et al., 2007; Weisberg et al., 2007; Bellebaum et al., 2013) have consistently confirmed the importance of previous experience in the learning-based formation of the cortical cell assemblies that subsume the cortical representation of concepts.

In the data suggesting the existence of endogenous constraints, the most important come from work (Mahon et al., 2009) indicating that the medial-to-lateral bias in category preferences for artifacts vs. animals, which is present in the ventral surface of the temporo-occipital cortex in sighted individuals, is also present in congenitally blind subjects. According to Mahon and Caramazza (2011), this observation suggests that, if visual experience is not necessary for the emergence of a categorical neural organization, then innate patterns of connectivity must mediate the integration of information critical for the organization of each category.

More inconclusive, though mainly supporting the experience-dependent hypothesis, is the fact that the anatomical structures disrupted in a given type of category-specific semantic disorder correspond to the areas of convergences of the sensory-motor information which has a major role in the construction of that category. This observation which (as we have seen in a previous section of this survey) was predicted by the “sensory-motor model of semantic knowledge”, is not necessarily at variance with an innatistic model, if we assume that the convergence of critical sensory-motor information in well defined cortical areas may have taken place in the course of evolution.

Therefore, even if the rationalist approach did not explicitely acknowledge that the empiricist approach was correct, it made similar predictions, leading to the conclusion that at the present state of knowledge it is impossible to choose between experience-dependent and inborn models of conceptual representations.

This interlocutory conclusion may be necessary because the question about experience-dependent vs. inborn semantic categories cannot be an either/or issue and both inborn features and experience shape semantic categories. Nevertheless, the weight of these complementary determinants of conceptual representations has yet to be explored. For instance, a recent study of genetic and environmental influences on the VWFA and the fusiform face area (FFA) has shown that activation of the VWFA is partially under genetic control, whereas activation of the FFA is primarily influenced by individual experience (Pinel et al., 2014). These results are obviously at variance with those obtained by Striem-Amit et al. (2012), which are reported in Section “Data Suggesting the Innate Nature of the Categorical Brain Organization and Objections Raised to the Hypothesis that Innate Connectivity Patterns May Underlie Categorical Organization”, in a discussion of the data that support the innate nature of categorical brain organization.

In my opinion, it is likely that only some basic mechanisms, linked to the general architecture of the brain and to the connections between structures processing perceptual and action-related information are inborn and that more specific contents of knowledge is experience-dependent. On the other hand, since conceptual categories are constructed on the basis of cortical areas that process perceptual and action-related information and their interconnections, it is not easy to disentangle the innate processing systems from the experience-dependent contents of their joint action. It is only possible to highlight the results of studies that have consistently confirmed the importance of previous experience in the learning-based formation of cortical cell assemblies, which subsume the cortical representation of previously familiar or newly learned concepts.

In any case, even if both inborn features and experience shape semantic categories, the question of the relative contribution of novel information and of predefined structures (i.e., specific brain networks) remains unresolved. It also remains unclear whether any pre-quantitative model can determine the relative contribution of inborn and experience-dependent sources of influence (Hofmann and Jacobs, 2014).

## Future research directions

Since I indicated in the foregoing tentative concluding remarks that the question about experience-dependent vs. inborn semantic categories cannot be an either/or issue and that both inborn features and experience shape semantic categories, I feel it is necessary to indicate some research directions in the fields of Artificial Intelligence and genomic imaging, which might provide ways to address these issues in the future. As to the first direction, a pioneering study by Mitchell et al. (2008) has shown that brain activation in response to a line drawing of a concrete concept and the corresponding name can be predicted if semantic feature values for that concept and the patterns of brain activation for other concepts are given. Since then, multivariate pattern analysis (MVPA) has become an inductive method for determining brain sites that selectively respond to particular semantic features and these analyses are becoming more and more category-specific. Pereira et al. (2013), for instance, used a corpus of a few thousand Wikipedia articles about concrete high-imageability concepts to produce a low-dimensional semantic feature representation of those concepts. They showed that these features can be used to uncover similarity relations in brain activation for different concepts, which parallel relations in behavioral data from human subjects. If strong similarities could be uncovered between sighted and congenitally blind individuals in the patterns of brain activation for different abstract and concrete, high-imageability and low-imageability words, this could strengthen the inborn models of conceptual representation. As for the second direction, it can be argued that if genes determine functional imaging (e.g., Muñoz et al., 2009), this might help to illuminate the degree to which semantic category development is genetically pre-determined. For example, to further explore the finding that higher-order semantic impairments are implicated in formal thought disorders (FTD) in schizophrenia (e.g., Dwyer et al., 2014), Nicodemus et al. (2014) combined a computational linguistic approach with a candidate gene approach to examine the genetic architecture of a category fluency task in patients with schizophrenia. It is, therefore, possible that the weight of inborn and experience-dependent factors in the construction of conceptual categories will be clarified by these and other, similar research directions.

## Conflict of interest statement

The author declares that the research was conducted in the absence of any commercial or financial relationships that could be construed as a potential conflict of interest.
